# The effects of occupational exposure to manganese fume on neurobehavioral and neurocognitive functions: An analytical cross-sectional study among welders

**DOI:** 10.17179/excli2019-2042

**Published:** 2020-03-13

**Authors:** Younes Mehrifar, Mahshid Bahrami, Esmail Sidabadi, Hamideh Pirami

**Affiliations:** 1 Department of Occupational Health Engineering, School of Health, Isfahan University of Medical Sciences, Isfahan, Iran; 2 Department of Occupational Health Engineering, School of Health, Tehran University of Medical Sciences, Tehran, Iran; 3 Department of Occupational Health Engineering, Islamic Azad University, Sabzevar, Iran; 4 Department of Occupational Health, Faculty of Medical Sciences, Tarbiat Modares University, Tehran, Iran

**Keywords:** welders, manganese fume, blood manganese, Stroop test, Continuous Performance Test

## Abstract

This study aimed to measure concentrations of manganese fume in breathing zone (BZ) and blood among welders to assess neurocognitive and neurobehavioral functions among them. In this study 38 welders and 27 administrative employees participated. Q16 questionnaire was used to evaluate neurobehavioral symptoms. The computerized Stroop test and Continuous Performance Test (CPT) were used to assess neurocognitive functions. Sampling and analysis of manganese fumes in the BZ and blood samples were performed according to NIOSH-7300 and NIOSH-8005 methods, respectively. Average concentration of manganese in the welders' BZ and blood was 0.81 ± 0.21 mg/m^3 ^and 18.33 ± 5.84 µg/l. Frequency of neurobehavioral symptoms was significantly higher in welders compared with control group. Spearman correlation test showed a moderate correlation between Mn concentrations in the BZ and blood Mn levels (r_s_ = 0.352). There were statistical moderate and strong correlations between the frequency of neurobehavioral symptoms and manganese concentrations in the BZ (r=0.504) and blood Mn levels (r=0.643).The Pearson correlation coefficient (r=0.433-0.690) obtained on the psychological tests showed a moderate to strong correlation between manganese concentrations in the welders' BZ and blood and some indices of the Stroop test and CPT**. **The results of this study can confirm the effect of manganese inhalation on creating neurobehavioral and neurocognitive impairments in welders.

## Background

Welding is the process of joining metallic components by heating up to the proper temperature with or without a filler metal (Karimi Zeverdegani et al., 2017[54]). Welding fumes have the greatest health hazard compared with other harmful factors (gases, noise, heat, and UV) (Mulyana et al., 2016[68]; Rahmani et al., 2016[79]). According to statistics, there are 500,000 full-time welders in the United States (Mehrifar et al., 2018[66]) and 5.5 million welding-related jobs in Europe (Amza et al., 2013[3]). The American Conference of Governmental Industrial Hygienists (ACGIH) has reported the recommended time-weighted average (TLV-TWA) to be 5 mg/m^3^ and 0.02 mg/m^3^ for fumes and manganese, respectively (ACGIH, 2017[1]). Most studies on welding fumes have focused on their respiratory effects and reported airway cells stimulation, reduced pulmonary function, asthma, bronchitis, pneumoconiosis, or lung cancer (Antonini, 2003[4]). There is little information on non-pulmonary effects of welding fumes, especially their neurocognitive effects (Antonini et al., 2012[7]). Welders are the largest working group exposed to manganese. Particles smaller than 1 µm are produced by welding which can easily penetrate pulmonary alveoli (Antonini et al., 2009[5]; Berlinger et al., 2011[11]). Recent studies have shown the effect of manganese on the development of neurological disorders among welders (Flynn and Susi, 2009[36]). However, manganese is a vital element in the human body, over-exposure and inhalation of it can produce neurological effects (Curran et al., 2009[28]). Welding fumes containing manganese cause magnetic field heterogeneity and increase rotation of brain protons which will result in shortening the proton relaxation times (Haacke et al., 2005[44]). Manganese accumulates in the brain and can cause central nervous system disorders as well as psychiatric disorders (Antonini et al., 2006[6]). Cognitive-neurological deficits associated with Mn exposure occur more often in the cerebral cortex, especially in the anterior cingulate cortex. Neuroimaging studies also focus on this part of the brain (Ma et al., 2018[62]). Bowler et al. reported high incidence rates of neurological symptoms of manganism as well as disorders such as anxiety, depression and confusion among welders (Bowler et al., 2006[14]). Long et al. used magnetic resonance imaging (MRI) to assess manganese accumulation in welders’ brains in machine-building and iron melting plants. The results of this study indicated higher levels of manganese in the anterior cingulate cortex, hippocampus and thalamus of fume-exposed welders compared with those in the control group (Long et al., 2014[59]). 

Both occupational and environmental exposures to manganese have been associated with neuropsychological function impairment (Hudnell, 1999[50]; Levy and Nassetta, 2003[58]; Pal et al., 1999[74]; Santamaria et al., 2007[80]; Zoni et al., 2007[96]). Welders make up one of the largest occupational groups studied for the health effects of Mn due to easy access and their inhalation exposure to Mn-containing particles (Bowler et al., 2007[17]). Welders’ exposure to manganese (Mn) seems to affect both psychomotor and cognitive functions, especially executive functions, concentration maintenance, cognitive flexibility, oral learning and working memory (Bowler et al., 2007[17]; Bowler and Lezak, 2015[16]). Studies on non-human mammals support the neurological effects of chronic exposure to Mn, and human findings indicate impaired fine motor skills, attention and concentration, working memory, visual perception and frontal lobe and parietal lobe disorders (the primary site for Mn accumulation in addition to the basal ganglia) (Burton and Guilarte, 2008[19]; Guilarte et al., 2006[43], 2008[42]; Guilarte, 2013[41]; Schneider et al., 2006[83], 2013[84], 2015[85]). Few studies have investigated the association between neuropsychological disorders and Mn deposition in certain brain regions (Schneider et al., 2006[83], 2013[84], 2015[85]).

The brain is sensitive to excess Mn and high levels of Mn accumulation can cause known neurodegenerative diseases, such as "manganism". This disease is characterized by symptoms like those of Parkinson’s disease such as motor dysfunctions including tremor, rigidity, dystonia and/or ataxia as well as psychiatric disorders such as irritability, obsessive-compulsive disorder, hallucinations and also cognitive impairments such as memory deficits, reduced ability to learn and reduced cognitive flexibility (Levy and Nassetta, 2003[58]; Josephs et al., 2005[52]).

Studies show that welders’ exposure to high concentrations of manganese-containing welding fumes will lead to increased prevalence of neurobehavioral symptoms among them (Antonini et al., 2006[6]; Wastensson et al., 2012[92]; McMillan, 1999[63]; Hassani et al., 2016[49]; Wang et al., 2006[91]; Ellingsen et al., 2008[32]; Cowan et al., 2009[26]). Cross-sectional studies have shown impaired motor function (Bowler et al., 2006[14]; Ellingsen et al., 2008[32]; Sjögren et al., 1996[87]; Bowler et al., 2003[13]; Chang et al., 2009[22]; Laohaudomchok et al., 2011[56]; Ellingsen et al., 2013[33]) and impaired cognitive function (Bowler et al., 2003[13]; Chang et al., 2009[22]; Laohaudomchok et al., 2011[55]; Yuan et al., 2006[95]; Wang et al., 2006[91]; Bowler et al., 2006[14]). Several studies have shown a dose-response association between Mn-exposure and impaired function in cognitive domains such as attention, concentration, working memory, cognitive flexibility, diverse skills, digital span, and coding tasks (Bowler et al., 2007[17][18], 2015[15]; Ellingsen et al., 2008[32]; Lucchini et al., 1999[60]). 

In a study conducted by Park et al. on 48 welders in San Francisco Bay Area, the welders obtained low scores on the tests measuring planning, organizational skills, problem-solving strategies, perceptual and motor functions, memory, concentration, attention, and the ability to work with numbers (Park et al., 2006[76]). Chang et al. reported certain cognitive impairments in welders, including low memory test scores and deficits in fine motor skills (Chang et al., 2010[23][24], 2013[21]).

In Iran, few studies have been conducted on cognitive functions among manganese-exposed workers, especially welders. Since welding is used in all industrial and manufacturing centers, this study aimed to investigate the neurobehavioral and neurocognitive functions of welders working in a metal construct Manufacturer Company via breathing zone air monitoring, biological monitoring of blood and using standard and valid tests. 

## Methods

### Participants 

This analytical cross-sectional study was carried out in a metal construct manufacturer company in Iran. After a preliminary review of the industry, shielded metal arc welding (SMAW) was chosen as having major welding fume emissions. Using statistical analysis, 38 male welders (who met the inclusion criteria) were selected as the exposed groups and 27 male administrative employees were selected as the control group. The inclusion criteria were: no history of alcohol and tobacco use, no history of cardiac medications, blood glucose-lowering drugs, antidepressants and sedative drugs, anti-histamines, anti-Parkinson drugs and other medications, absence of color blindness, having normal hearing, having no history of cardiovascular diseases, respiratory problems and sleep disorders, previous or current psychiatric and neurological disorders and head injuries, no learning disability and having performed welding 3 hours a day on average for at least 6 months (Ghanadzadeh et al., 2009[38]). All these inclusion criteria were considered for the employees of the department of administrative services except welding experience. In this study, the prevalence of neurobehavioral symptoms, selective attention, and sustained attention were assessed using the Q16 questionnaire, computerized Stroop test and Computerized Continuous Performance test, respectively. Firstly, the demographic data were recorded and the study participants were assured that their private information would remain confidential.

### Data collection tools

#### Demographic and practice characteristics questionnaire

Personal characteristics such as age, height, weight, BMI and work experience related to the current occupation were studied using this questionnaire.

### Sampling and analysis of pollutants

#### Workplace air sampling

Breathing zone air sampling for manganese fumes was conducted at welding stations according to NIOSH 7300 (NIOSH, 2003[70]) using a personal sampler pump (224 PCMTX8; SKC, USA), calibrated with a digital calibrator (Defender-510, Canada), as well as Filter holders and Mixed cellulose esters filters (MCE) with 37 mm diameters, 0.8 µm pore sizes and 1-4 (L/min) air flow rates (NIOSH, 2003[70]). Additionally, breathing zone air sampling for total fumes was performed according to NIOSH 0500 (NIOSH, 1994[72]) using 37 mm (0.5 micron) PVC (Polyvinylchloride) preweighed filters with 1- 2 l (L/min/cm^2^) air flow rates. Additionally, air sampling was carried out in the workplace of the administrative staff to ensure lack of their exposure to Mn. In addition, for each 10 samples, a control sample was also prepared (Giahi et al., 2014[39]). During air sampling, ventilation and personal respiratory protective equipment were also taken into consideration.

#### Determination of welding total fume and manganese concentrations.

In order to determine manganese concentrations of welding fumes, the samples collected on filters (MCE) after extraction, based on NIOSH 7300, were analyzed using Varian Liberty RL inductively coupled plasma atomic emission spectroscopy (ICP-AES), Italy (NIOSH, 2003[70]). The total fume concentrations were determined before and after sampling according to NIOSH 0500 using weight differences between 37 mm (0.5 micron) Polyvinylchloride (PVC) filters.

#### Blood sampling and sample analysis 

Blood samples were drawn from all the participants, at the end of their working shifts, by an experienced expert of the medical diagnostic laboratory (according to the standard protocols). The samples were prepared according to NIOSH 8005 and the concentrations of their metal contents were determined by ICP-AES (NIOSH, 1994[71]).

### Neurobehavioral symptoms

#### Neurobehavioral symptom questionnaire 16 (Q16)

In this study, the Q16 questionnaire was used to assess neurobehavioral symptoms among the welders (Lundberg et al., 1997[61]). The Q16 questionnaire contains short questions with yes/no response alternatives on abnormal fatigue, tingling sensation, irritation and depression without any particular reason, difficulty focusing, etc. (Hassani et al., 2016[49]). Although the Q16 questionnaire was developed to monitor the effects of exposure to organic solvents on the central nervous system among workers, the benefits of using it for exposure to other neurotoxic agents have been proven (Bolla et al., 1995[12]). In Iran, Hassani et al. used this questionnaire to assess neurobehavioral symptoms among welders (Hassani et al., 2016[49], 2013[48]).

### Neurocognitive tests 

#### Computerized Stroop test

The Stroop test was first designed by Ridley Stroop to evaluate selective attention and cognitive flexibility (Stroop, 1935[90]). This test has been used in several studies to measure response inhibition ability, selective attention, cognitive variability and cognitive flexibility. The test consists of two steps. The first step is naming colors, and the goal of this step is only to practice and identify colors and place of keys on the keyboard; therefore, it does not affect the final result. The second step is the main implementation step in which 48 congruent and 48 incongruent combinations of four color words (red, blue, yellow and green) were presented. Congruent combinations are those in which the meaning of the word matches the color. Incongruent combinations are those in which color and word differ. The subject is required to identify the color of the word as quickly as possible, regardless of its meaning, based on the labels on the keyboard. Each stimulus is displayed on the screen for 2 seconds and the interstimulus interval is 800 thousandth of a second. Researchers believe that the color and word task in the second step of the test measures mental flexibility, response inhibition and interference (Wecker et al., 2000[93]). Ghadiri et al. reported the test-retest reliability coefficients to be 0.55 and 0.6 for the number of errors and the reaction time at the first step, respectively. Also, at second step, the reliability coefficient was 0.79 and 0.97 for the number of errors and for the reaction time, respectively (Ghadiri et al., 2006[37]). Based on Gulen's studies, test-retest reliability and concurrent validity of this test was 0.81 and 0.74, respectively (Golden, 1978).

#### Computerized Continuous Performance Test (CPT)

CPT measures sustained attention and impulsiveness or impulse control. On this test, the participant should pay attention to a set of relatively simple visual or auditory stimuli for a while (only visual stimuli are presented in this test) and give a key press response to the target stimulus. On this test, a total of 150 stimuli are presented. Each stimulus is presented for 200 thousandth of a second and the interstimulus interval is one second. The variables measured via this test are: a) omission errors (the participant fails to respond to a target stimulus indicating his/her difficulty inferring the stimulus). This kind of error is due to having trouble with concentration and *maintaining attention* that is an indicative of inattention to stimuli. b) Response errors (the participant responds to a non-target stimulus). This type of response error represents a failure of inhibitory control and is interpreted as impulsivity. c) The number of correct answers; and d) reaction. Hadianfard et al. (2001[45]) reported the test-retest reliability coefficients of this test to be within the range of 0.59- 0.93 for different parts of the test according to Karimi Aliabad et al. (2010[53]).

### Statistical analyses

The collected data were analyzed using SPSS21 software. Firstly, the normality of data distribution was detected using the Kolmogorov-Smirnov test. The mean and standard deviation were used to interpret descriptive statistics and T-test was used to interpret analytical statistics of normally distributed data. Spearman correlation and Pearson correlation tests was used to assess the association between two variables and a significance level of 0.05 was used.

## Results

This study was carried out on 38 welders and 27 employees of the department of administrative services in a metal construct Manufacturer Company. The mean age of the exposed group, with 12.58 ± 3.71 years of work experience, was 34.52 ± 11.24 years and the mean age of the control group, with 10.72 ± 4.51 years of work experience, was 36.25 ± 9.33 years. There was no significant difference between the two groups in their mean age, height, weight, and work experience. Some demographic and occupational data are presented in Table 1(Tab. 1). 

The results of the sampling of welders' breathing zone air showed that the average concentrations of manganese and total fume were 0.08 ± 0.02 mg/m^3^ and 9.08 ± 3.71 mg/m^3^, respectively (Table 2(Tab. 2)). The average concentrations of manganese and total fume in welding stations were significantly higher than the TLV-TWA recommended by the American Conference of Governmental Industrial Hygienists (ACGIH) for manganese (0.02 mg/m^3^) and total fume (5 mg/m^3^). Analysis of blood samples showed a significant increase in the average blood concentration of manganese in the welders (18.33 ± 5.84 µg/L) compared with those in the control group (10.24 ± 3.22 µg/L) (P <0.05). On the other hand, a significant increase was observed in the frequency of neurobehavioral symptoms among the exposed welders (6.32 ± 2.83) compared with that in the control group (1.55 ± 0.61) (P <0.05) (Table 2(Tab. 2)).

The Stroop test indices for the exposed and control groups are presented in Table 3(Tab. 3). The findings of the study showed that there was a significant reduction in the welders' mean scores for accuracy (number of correct answers) compared with those of the control group (P <0.05). On the other hand, there was a statistically significant increase in the welders' mean scores for speed (mean reaction time in correct response to a stimulus (milliseconds) compared with those of the control group (P <0.05).

The findings of the continuous performance test showed significant increases in the numbers of omission errors, response errors and reaction times among the welders compared with those in the control group (P <0.05). On the other hand, a significant decrease was observed in the number of correct responses given by the welders compared with those given by the control group (P <0.05) (Table 4(Tab. 4)).

The results of Spearman correlation test showed a moderate and significant correlation between manganese concentrations in the breathing zone and blood Mn concentrations (r_s_ = 0.352 ,P-value = 0.02) in the exposed group.

The results also showed statistically significant moderate and strong correlations between the frequency of neurobehavioral symptoms (Q16) and the breathing zone Mn concentrations (r = 0.504) and blood (r = 0.643) Mn concentrations, respectively in the exposed group (Table 5(Tab. 5)).

The results of Pearson correlation test showed a statistically significant direct strong correlation between breathing zone and blood Mn concentrations and Stroop test indices including the mean reaction time in the congruent conditions (r_Mn air_=0.620) (r_Mn B_=0.688) and in the incongruent conditions (r_Mn air_=0.646) (r_Mn B_=0.602) in the exposed group. On the other hand, there was a significant moderate and inverse correlation between the Stroop test indices of correct responses in the congruent and incongruent conditions and Mn concentrations in the welders' breathing zone and blood (Table 5(Tab. 5)).

Also, the results in Table 5(Tab. 5) show a statistically significant direct moderate correlation between breathing zone and blood Mn concentrations and the indices of the continuous performance test including omission errors and response errors. Additionally, a statistically significant direct strong correlation was found between breathing zone and blood Mn concentrations and the reaction time (r_Mn air_=0.690) (r_Mn B_=0.612). On the other hand, a moderate inverse correlation was found between the number of correct responses and breathing zone and blood Mn concentrations in the exposed group (Table 5(Tab. 5)).

## Discussion

Chronic exposure to manganese (Mn) is a health concern in occupations such as welding due to its proven stimulatory effects on basal ganglia disorders (Al-Lozi et al., 2017[2]). In this study, some cognitive functions (selective and sustained attention) and neurobehavioral symptoms appearing after chronic exposure to manganese were examined in welders. Our findings showed that the average concentrations of Mn in the welders' breathing zone was 0.08 ± 0.02 mg/m^3^, which was significantly higher than the standard exposure limit (TLV-TWA) (0.02 mg/m^3^) recommended by ACGIH for manganese. This finding is consistent with previous studies of the author (Mehrifar et al., 2018[64], 2019[65]). Also, the average concentrations of Mn in the welders’ blood were higher than the normal reference ranges for blood Mn (O’Neal and Zheng, 2015[73]). High concentrations of blood manganese result from lack of use of respiratory protective equipment by most welders, prolonged exposure to welding fumes, and lack of adequate ventilation in their workplace. 

In this study, Q16 questionnaire was used to evaluate neurobehavioral symptoms of welders exposed to manganese as a neurotoxic chemical. However, this questionnaire has been designed to investigate neurobehavioral symptoms of workers exposed to organic solvents (Lundberg et al., 1997[61]), some studies have used this tool to investigate neurobehavioral symptoms of welders (Sjögren et al., 1990[86]; Laohaudomchok et al., 2011[56]; Sriram et al., 2010[88]).

Some studies have reported Mn exposure threshold for the incidence of subclinical neurological effects to be 0.27-1.7 mg/m^3^ (Deschamps et al., 2001[29]; Clewell et al., 2003[25]; Myers et al., 2003[69]). In the present study, the average concentrations of Mn in the breathing zone were within the mentioned range, and a significant increase was observed in neurobehavioral symptoms among the welders compared with those of the control group, which is consistent with the results of previous studies (Hassani et al., 2016[49], 2013[48]; Ellingsen et al., 2008[32]; Bowler et al., 2003[13]; Sjögren et al., 1990[86]).

Similar results have been obtained in other studies. A study conducted by Hassani et al. (2013[48]) in a steel industry entitled "A survey of neurobehavioral symptoms of welders exposed to manganese" showed that blood concentrations of Mn were significantly higher in welders compared with those in the administrative staff. Also, the frequency of neurobehavioral symptoms of the welders exposed to manganese was higher than that of the administrative staff; however, the authors observed no significant correlation between air Mn concentrations and neurobehavioral effects (Hassani et al., 2013[48]). The results of our study showed a significant association between neurobehavioral effects and air and blood concentrations of Mn, but this difference between the findings is justified since Hassani et al. reported manganese concentrations in air (0.023 ± 0.012 mg/m^3^) to be within permissible limits. The results of studies conducted by Lee et al. (2017[57]), Park and Berg (2018[75]) and Ellingsen et al. (2015[30]) showed that the frequency of neurological symptoms in welders was significantly higher compared with that in the administrative staff. The study conducted by Baker et al. showed that the exposure of amateur welders to metal fumes, even below the ACGIH permissible exposure limit, significantly reduced the spin-net relaxation time (T1) in anterior and posterior cortex and basal ganglia (Baker et al., 2015[8]).

The results of this study showed a moderate and significant correlation between breathing zone Mn concentrations and blood Mn concentrations in welders. Long et al. conducted a study on 40 welders and reported a positive and direct correlation between breathing zone Mn concentrations and blood Mn concentrations in both exposure and control groups (Long et al., 2014[59]). On the other hand, Ellingsen et al. (2006[31]), Pesch et al. (2012[77]) and Halatek et al. (2005[46]) have suggested that there is a low correlation between breathing zone Mn concentrations and blood Mn concentrations in welders. 

The Stroop test analysis showed that welders had less response inhibition ability and selective attention compared with the control group. Response inhibition and selective attention have congruent and incongruent components. The exposed group gave a lower percent of correct responses in longer reaction times in both congruent and incongruent conditions compared with the control group. This finding suggests that the exposed group had less response inhibition ability and selective attention. People are expected to demonstrate longer response time for the incongruent condition: The less the difference between response time for these two conditions, the better (in case of a large number of correct responses). The exposed group demonstrated longer response time for the incongruent condition compared with that for the congruent condition, while the number of correct answers was reduced since they had less response inhibition ability and selective attention.

In the present study, the welders’ scores on Stroop indices decreased significantly compared with those of the control group and this finding is consistent with the results of previous studies (Bowler et al., 2006[17], 2007[18]; Chang et al.,2013[21]). Bowler showed a significant decrease in the scores on the Stroop color and word test and working memory indices among the welders with an average of 24.9 years welding experience in open workplaces (Bowler et al., 2003[13]). In Park’s study, the welders obtained low scores on Rey-Osterrieth Complex Figure Test, Working Memory Index, Stroop Color and Word Test, and Auditory Consonant Trigram Test which measure planning, organizational skills, problem-solving strategies, motor and perceptual functions, memory, concentration, attention, and ability to work with numbers (Park et al., 2006[76]).

Chang et al. investigated volume changes of globus pallidus and cerebellum in welders chronically exposed to Mn and examined the neurobehavioral tests. Their results showed a significant decrease in the volume of these areas of the welders’ brains, and in their scores on Stroop indices compared with those of the control group. There was also a correlation between brain volume decline and neurobehavioral, cognitive and motion impairments. This study showed a significant increase in blood manganese level and Pallidal index in welders compared with those in the control group (Chang et al., 2013[21]). Another study showed decreased memory functions among welders compared with those in the control group. Also, the results of MRI showed increased brain network activity during working memory tasks among Mn-exposed welders compared with those in the control group (Chang et al., 2010[23]). Sustained attention refers to the ability to maintain attentional focus on a particular subject. "Attention" can be defined regarding the number of errors in test taking. Thus, the greater an individual’s attention during test taking, the lower the number of errors, and vice versa. Unfortunately, few studies have investigated sustained attention in welders exposed to welding fumes, especially manganese fumes. The present study showed a significant increase in the number of omission errors and response errors among welders compared with those of the control group (P <0.05), which indicates lack of attention in the exposed group. 

On the other hand, attention is closely associated with reaction time. Thus, the greater the attention of participants during test taking, the shorter their reaction time, and vice versa: the lower the level of attention, the longer the reaction time. Moreover, average reaction time is associated with the speed of information processing. Reaction time is actually the elapsed time between the presentation of a sensory stimulus and the subsequent behavioral response (Jensen, 2006[51]). Reaction time varies depending on the type of activity, attention and awareness of the situation. In emergency situations, people may have neural responses or even avoid responding to stimuli (Peters and Peters, 2006[78]). In other words, reaction time is the time it takes a person to perceive the situation and process a response (Stranks, 2007[89]). This study showed a significant increase in the reaction time of the group of welders compared with that of the control group (P <0.05). Our Stroop test showed this increased reaction time in the exposed group. On the other hand, the number of correct responses was significantly reduced in the exposed group compared with that in the control group. Some studies have also reported increased reaction times even during exposures to very low concentrations of manganese; like Laohaudomchok et al. who investigated neuropsychological effects of manganese exposure on welders exposed to low concentrations of manganese (12 µg/m^3^). The results of this study showed a significant association between increased cumulative Mn exposure index (CEI) and slowed reaction time on the Continuous Performance Test. In this study, the total CEI was calculated based on the welders' past exposure, including the time taken to perform specific tasks, the average concentration of manganese and the percentage of times using a respirator (Laohaudomchok et al., 2011[56]). Bast-Pettersen et al. investigated neurobehavioral functions among aluminum welders. They showed that the welders’ reaction times were faster on the Continuous Performance Test compared with those in the control group. Although this difference was not statistically significant, it was associated with aluminum concentration in air (Bast-Pettersen et al., 2000[10]). Symptoms of central nervous system disorders are common among the welders exposed to aluminum for a long time. The most important symptoms reported among aluminum-exposed welders include fatigue, distraction, depression and memory deficits (Sjögren et al., 1996[87], 1990[86]; Hänninen et al., 1994[47]).

In a meta-analysis study performed by Meyer-Baron et al. Results showed a decrease in cognitive and motor performance scores in workers exposed to manganese compared to reference workers (Meyer-Baron et al., 2013[67]).

A study by Ferreira (2010[34]) on 14 welders showed a statistical moderate and positive relationship between Mn concentrations in the breathing zone and the number of hand errors, reduced finger skills, and reduced eye-hand coordination on the Perdue pegboard and mirror drawing tests (Ferreira, 2012[34]).

It is worth noting that this study had limitations that may affect the results. First, we did not have any information on the workers’ past exposure to manganese, since a systematic air quality monitoring network was not available. There was also no documentary information on the efficiency of the ventilation system. Second, due to limitations caused by the lack of cooperation and lack of access to IQ documentation of the subjects under study the expression of IQ values in the study was not possible. In this study, we did not measure the level of education of participants as a confounding factor in test results. However, studies reported that the correlation between years of education and intellectual capacity is for example unsatisfactory (Cervilla et al., 2000[20]; Schmand et al., 1997[81]).

Third, blood manganese levels show recent exposures and are not reliable for determining long-term exposures (Wongwit et al., 2004[94]; Baker et al., 2014[9]; Crossgrove and Zheng, 2004[27]). Studies show that Pallidal Index (PI) is associated with cognitive impairment, so Pallidal Index may be a better indicator of cognitive ability compared with blood (Chang et al., 2009[22]). Fourth, welders are exposed to numerous processes, ergonomic and environmental problems, gases, fumes, and multiple neurochemicals during welding (Finley and Santamaria, 2005[35]). Hence, the cognitive impairments observed among the welders cannot be solely due to manganese exposure. 

## Conclusion

Welding is one of the most important occupations in most industries, producing a wide range of harmful gases and fumes. The welders’ breathing zone and blood sampling indicated that welders are exposed to high levels of total fume and manganese-containing fumes. Our results showed that exposure to high levels of manganese fumes will result in reduced attention, increased reaction time and neurobehavioral and neuropsychological disorders in welders.

The purpose of using several different tests to assess psychological effects was to consider most of the factors related to the efficiency of the neurobehavioral system performance in the study subjects to arrive at an accurate and comprehensive output to comment on the consequences of exposure to toxic fumes containing manganese in the workplace.

Therefore, in order to keep welders healthy, control actions should be taken for this pollutant, as soon as possible. It is hoped that the present study will provide a framework for other scholars to look beyond the neurological and psychological effects of manganese as a vital element in welding process.

## Competing interests

The authors have no competing interests to declare. 

## Author contribution

HP managed and planned the project. YM and HP analyzed the data and made the preliminary information in a state of measurable and MB,YM, ES and HP were a major contributor in writing the manuscript. HP collected the data in the field. All authors read and approved the final manuscript.

## Conflicts of interest

All authors declare no conflict of interest.

## Acknowledgement

Authors thank the management and all of the welders who helped us conducting the present study.

This research did not receive any specific grant from funding agencies in the public, commercial, or not-for-profit sectors.

## Figures and Tables

**Table 1:**
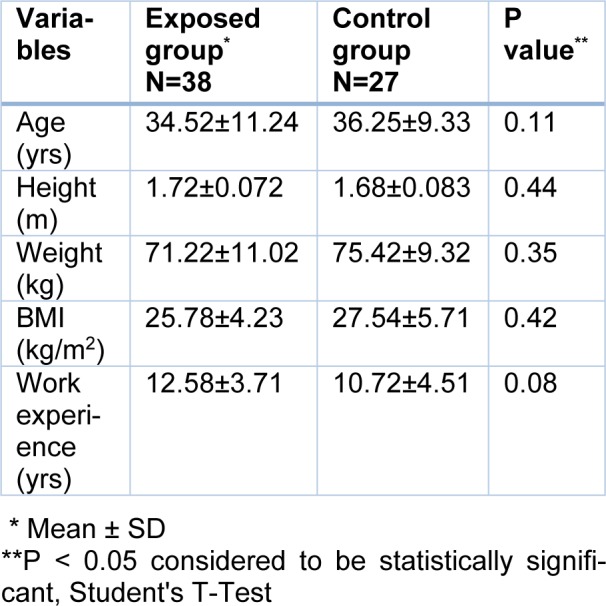
Characteristics of the exposed and control groups

**Table 2:**
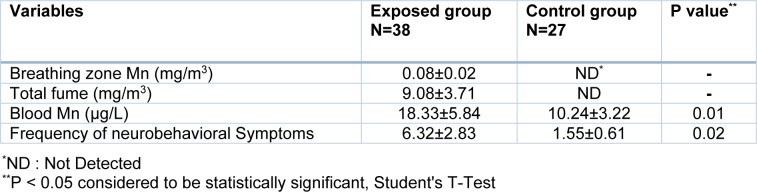
Mean (SD) concentrations of manganese fume in breathing zone, blood and frequency of neurobehavioral symptoms in exposed and control groups

**Table 3:**
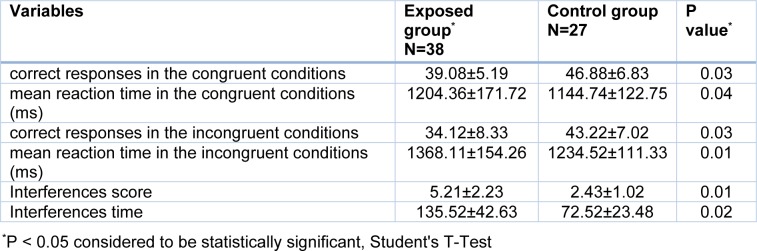
Comparison of mean and standard deviation of scores of Stroop test indicators in exposed and control groups

**Table 4:**
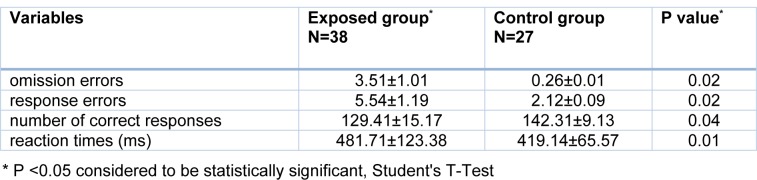
Comparison of mean and standard deviation of scores of continuous performance test indicators in exposed and control groups

**Table 5:**
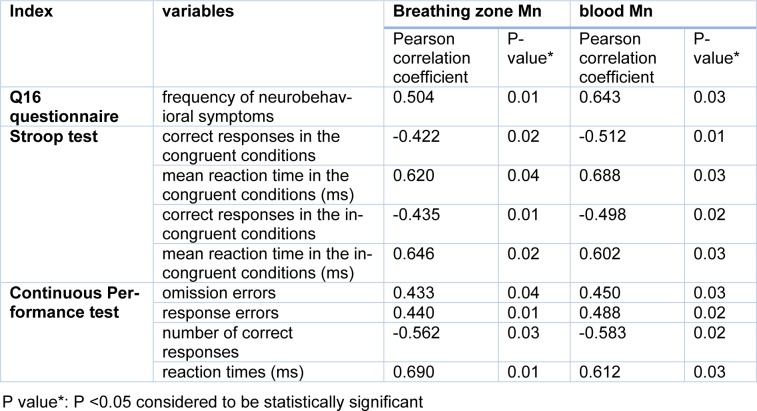
Correlation between manganese concentration of breathing zone and blood with neurobehavioral and neurocognitive symptoms in welders (n = 38)
